# Evidence for Pervasive Adaptive Protein Evolution in Wild Mice

**DOI:** 10.1371/journal.pgen.1000825

**Published:** 2010-01-22

**Authors:** Daniel L. Halligan, Fiona Oliver, Adam Eyre-Walker, Bettina Harr, Peter D. Keightley

**Affiliations:** 1 Institute of Evolutionary Biology, School of Biological Sciences, University of Edinburgh, Edinburgh, United Kingdom; 2 Centre for the Study of Evolution and School of Life Sciences, University of Sussex, Brighton, United Kingdom; 3 Max-Planck-Institute for Evolutionary Biology, Plön, Germany; University of Arizona, United States of America

## Abstract

The relative contributions of neutral and adaptive substitutions to molecular evolution has been one of the most controversial issues in evolutionary biology for more than 40 years. The analysis of within-species nucleotide polymorphism and between-species divergence data supports a widespread role for adaptive protein evolution in certain taxa. For example, estimates of the proportion of adaptive amino acid substitutions (*α*) are 50% or more in enteric bacteria and Drosophila. In contrast, recent estimates of *α* for hominids have been at most 13%. Here, we estimate *α* for protein sequences of murid rodents based on nucleotide polymorphism data from multiple genes in a population of the house mouse subspecies *Mus musculus castaneus*, which inhabits the ancestral range of the Mus species complex and nucleotide divergence between *M. m. castaneus* and *M. famulus* or the rat. We estimate that 57% of amino acid substitutions in murids have been driven by positive selection. Hominids, therefore, are exceptional in having low apparent levels of adaptive protein evolution. The high frequency of adaptive amino acid substitutions in wild mice is consistent with their large effective population size, leading to effective natural selection at the molecular level. Effective natural selection also manifests itself as a paucity of effectively neutral nonsynonymous mutations in *M. m. castaneus* compared to humans.

## Author Summary

The prevalence of natural selection at the DNA level remains a controversial issue in evolutionary biology. In particular, estimates of the proportion of adaptive amino acid changes (*α*) vary greatly between taxa, being 50% or more in bacteria and fruit flies, but at most 13% in hominids. Here, we infer the frequencies of polymorphisms in protein-coding genes of 15 *Mus musculus castaneus* individuals sampled from the ancestral range of the house mouse species complex. By combining the polymorphism data with nucleotide divergence to the related murid species *M. famulus* and the rat, we obtain an estimate for *α* of 57%. This represents the first estimate of *α* for a mammal other than humans. The high rate of adaptive protein evolution in wild mice and other taxa implies that hominids may be somewhat unusual in having low rates of adaptive protein evolution. One possible cause of this is the low effective population size in humans, which is predicted to lead to less effective natural selection and fewer adaptive mutations. This is consistent with the higher frequency of nearly neutral deleterious amino acid mutations in hominids than murids that we infer in our analysis.

## Introduction

Several approaches have revealed evidence for adaptation at the molecular level. Local reductions in diversity, indicating selective sweeps, have been identified in populations of a number of species [1],[2]. In Drosophila, reductions in neutral diversity have also been associated with increased divergence at amino acid sites, indicative of recurrent selective sweeps of advantageous amino-acid changing substitutions [3]. There is also evidence for a general reduction in diversity close to conserved sequence features in protein-coding genes and noncoding elements of hominids [4]. These reductions can be attributed either to genetic hitchhiking of positively selected alleles or background selection against negatively selected alleles. Higher *F_ST_* within genic than nongenic regions of the human genome suggests that genic regions are subject to local adaptation within populations [5], (although see [6]). Further evidence for adaptation has come from attempts to identify sites or genes subject to recurrent positive selection by looking for an excess of substitutions in sites of interest over that expected. For example, a nonsynonymous to synonymous divergence ratio exceeding one at a locus may be evidence for positive selection at nonsynonymous sites. An approach that tests for an excess of substitutions at selected sites is the McDonald-Kreitman test [7], which contrasts levels of polymorphism with divergence at selected sites (e.g., nonsynonymous sites) and linked putatively neutral sites (e.g., synonymous sites). A recent extension of this test quantifies molecular adaptation as the fraction of substitutions driven to fixation by positive selection (*α*) [8],[9] by comparing the observed number of selected substitutions to the number expected, based on levels of polymorphism and divergence at neutral sites.

The application of derivatives of the McDonald-Kreitman test to amino-acid changing sites has resulted in a wide range of estimates of α, the causes of which may be multifaceted. Relatively high estimates of α have been obtained for enteric bacteria [10] and consistently high estimates have been obtained for Drosophila [9], [11]–[13], suggesting that α may be 50% or more in these species. On the other hand, estimates from yeast [14],[15], Arabidopsis [16] and hominids have been low. In the case of hominids, several independent estimates have found α for hominids to be 13% at most (with the exception of an estimate by Fay et al. [8]) [17]–[20].

Some of the observed variation in estimates of *α* may also be attributable to differences in the methods used. Specifically, some estimates of *α* are compromised by slightly deleterious mutations, since these contribute proportionately more than neutral polymorphisms to diversity than divergence. If slightly deleterious mutations are prevalent and not properly accounted for then *α* could be substantially underestimated [21]. This may partially explain low estimates of *α* obtained using methods that do not incorporate explicit population genetics models (e.g. yeast [14],[15] and Arabidopsis, [16]). Recently, improved methods to estimate *α* have been developed that model the contribution of slightly deleterious mutations to polymorphism and divergence [20],[22].

However, estimates of *α* obtained from hominids are low, even when based on methods that attempt to model for slightly deleterious mutations [20],[22], whereas estimates from Drosophila are high [22]. It is possible that this observation is a consequence of differences in effective population size (N*_e_*). *Drosophila melanogaster* is estimated to have an N*_e_* of 1–2 million, whereas hominids seem to have an unusually low recent N*_e_* [23]. In *D. miranda*, which is estimated to have a lower N*_e_* than *D. melanogaster*, N*_e_* is probably still an order of magnitude larger than that for hominids, and a high estimate of *α* was observed [13]. The proportion of adaptive substitutions is expected to depend on N*_e_* for two reasons. Firstly, a higher proportion of both advantageous and deleterious mutations are expected to be effectively neutral in species with low N*_e_*, because selection for/against slightly advantageous/deleterious mutations becomes less effective (see [24]). In such species, a higher proportion of slightly deleterious mutations, and conversely, a smaller proportion of slightly advantageous mutations, are expected to contribute to divergence. Secondly, if the rate of adaptation is limited by the supply of mutations, then species with low N*_e_* will adapt more slowly simply because they have to wait longer for each new advantageous mutation to appear in the population.

Currently there are no estimates for rates of adaptive evolution of protein-coding genes in mammals other than hominids, particularly for species with higher N*_e_*. *M. m. castaneus* populations from NW India, a region believed to be part of the ancestral range of the house mouse sub-species complex [25], have silent-site diversity for the X-chromosome of the order of 1% [26]. When combined with an estimate of the per nucleotide mutation rate per generation for murids [27], this level of diversity suggests that *M. m. castaneus *N*_e_* is two orders of magnitude higher than recent N*_e_* of hominids, and comparable to N*_e_* typically seen in Drosophila. We hypothesised that the murid protein-coding genes would therefore show pervasive natural selection, whereas its impact is much reduced in hominid orthologs. We tested the hypothesis by estimating *α* from protein-coding genes of murid rodents by comparing nucleotide polymorphism data of *M. m. castaneus* sampled from the NW Indian population with nucleotide divergence to *M. famulus* and the rat.

## Results/Discussion

**Figure 1:**
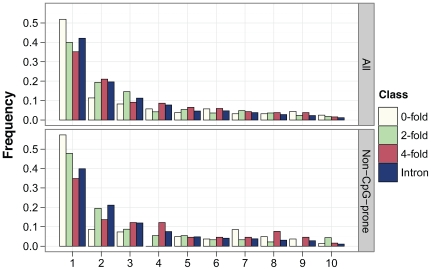
Plots of the site frequency spectra for 0-fold, 2-fold, and 4-fold degenerate and intronic sites. The upper plot includes all sites, whereas the lower plot is for non-CpG-prone sites only.

To infer levels of negative and positive selection in murid protein-coding genes, we analysed nucleotide diversity within a sample of 15 wild, unrelated *M. m. castaneus* from the NW Indian population together with the nucleotide divergence between *M. m. castaneus* and either *M. famulus* or the rat. We sequenced amplicons from a sample of 77 autosomal loci that are part of the Environmental Genome Project (EGP) [28] (details of the genes sequenced are presented in Table S1). These loci are not a random sample, since they are associated with human genetic diseases whose susceptibility is influenced by environmental challenge. However, they show low rates of adaptive amino acid substitution that are typical of hominids [17]–[20]. Summary statistics concerning nucleotide diversity at intronic, 4-fold degenerate, 2-fold degenerate and 0-fold degenerate sites are shown in Table 1 and Table S2 and the allele frequency distributions (or site frequency spectra, SFS) are plotted in Figure 1. As expected, zero-fold degenerate sites have the lowest nucleotide diversity, lowest divergence, the most negatively skewed SFS, and the most negative estimate of Tajima's D, a statistic related to the skew in the distribution of allele frequencies [29]. This is consistent with purifying selection keeping most amino acid mutations at low frequencies and reducing the number of fixations. Nucleotide diversity is higher for synonymous than intronic sites, as is Tajima's D. Together with a slightly higher synonymous than intronic divergence between *M. m. castaneus* and *M. famulus* (Table 1), this suggests somewhat weaker purifying selection acting on synonymous than intronic sites in murids, and that synonymous sites are likely to be the most appropriate neutral reference.

**Table 1:** Estimates of percentage diversity (*θ_π_* and *θ_S_*) summed over all sites for *M. m. castaneus*, and estimates of percentage divergence (*d*) to *M. famulus* or the rat.

Site class	*% θ_π_* [SE]	*% θ_S_* [SE]	Tajima's *D* [SE]	*% d* (*M. famulus*) [SE]	*% d* (rat) [SE]
0-fold	0.15 [0.019]	0.21 [0.019]	−0.87 [0.19]	0.79 [0.12]	3.5 [0.42]
2-fold	0.54 [0.053]	0.67 [0.053]	−0.72 [0.19]	2.3 [0.26]	12 [0.61]
4-fold	0.79 [0.086]	0.91 [0.086]	−0.49 [0.16]	3.3 [0.27]	19 [0.80]
Intron	0.66 [0.049]	0.83 [0.049]	−0.85 [0.095]	2.8 [0.15]	15 [0.49]

Standard errors are shown in square brackets.

Recent N*_e_* in wild mice, humans and Drosophila can be compared by equating synonymous site nucleotide diversity (*θ_π_*) to 4N*_e_μ*, where *μ* is an estimate of the mutation rate per site per generation (Table 2). Using estimates of *μ* based on synonymous site divergence and an assumption of two generations per year, our estimate of N*_e_* for wild mice of 580,000 is similar to that obtained for African *D. melanogaster*, whereas, in African populations of humans, N*_e_* is nearly two orders of magnitude smaller. Our estimate for *M. m. castaneus* is consistent with, although marginally higher than, a recent estimate of 400,000 (also assuming two generations per year) [30], based on smaller sample of loci. Nucleotide diversity in NW Indian *M. m. castaneus* is approximately one order of magnitude higher than observed in derived populations of *M. m. domesticus* and *M. m. musculus* from Europe and two orders of magnitude higher than among laboratory inbred mouse strains [26],[30],[31].

**Table 2:** Estimates of percentage nucleotide diversity (*θ_S_*) at 4-fold degenerate sites, the per nucleotide site mutation rate per generation (*μ*), and recent *N_e_* in *M. m. castaneus*, African humans, and African *D. melanogaster*.

Species	*% θ_π_*	Dataset for *θ_π_*	*μ*×10^−9^	Reference (for *μ*)	*N_e_*
*M. m. castaneus*	0.79	This study	3.4	[27]	580,000
Human	0.11	[28]	25	[52]	9,300
*D. melanogaster*	1.70	[11]	5.8	[53]	730,000

Estimates of *N_e_* are obtained assuming *θ_π_* = 4*N_e_μ*.

To estimate parameters of the distribution of fitness effects of deleterious amino acid-changing mutations we used a maximum likelihood (ML) procedure [32] that contrasts the SFS at putatively neutral sites (four-fold degenerate or intronic sites in this case) with sites assumed to be subject to purifying selection (nonsynonymous sites). The procedure fits a gamma distribution of deleterious mutational effects to the nonsynonymous SFS, and a demographic model to both the neutral and nonsynonymous SFSs that allows a step change in population size at some time in the past. The method assumes that positively selected mutations make a negligible contribution to polymorphism. Selective effects (*s*) of new amino acid mutations are estimated as the product of N*_e_* and *s* (see Materials and Methods for details of the method). Assuming four-fold sites as the neutral reference, estimates of proportions of amino acid mutations that have fitness effects in different N*_e_s* ranges under the best-fitting mutation effect distributions are compared in Table 3 for our *M. m. castaneus* data set, three African or African-American human data sets ([28]; the “Seattle SNPs” Programs for Genomic Applications (PGA) [33]; the dataset of Boyko et al. [20]) and an African *D. melanogaster* data set [11]. Similar results are obtained if intronic sites are used as the neutral reference (Table 3). Nearly neutral deleterious amino acid mutations (*i.e.*, mutations for which N*_e_s*<1), which have an appreciable chance of drifting to fixation, are relatively uncommon in both mice and Drosophila (10% and 6% of amino acid mutations, respectively), whereas they make up ∼20% of amino acid mutations in humans (maximum *P* = 0.038 for mouse versus human comparison, see Table 4 for details; *P* = 0.25 for mouse *vs.* Drosophila comparison). Strongly deleterious mutations (N*_e_s*>10), which essentially never become fixed, are inferred to be somewhat more frequent in mice and Drosophila (79% and 87%, respectively; *P* = 0.21) than humans (∼70%; *P*<0.05 for all contrasts with mice except one, see Table 4 for details). Whilst it is possible that these differences between the species in the relative frequencies of mutations in different N*_e_s* categories reflect differences in the distribution of absolute selection coefficients (*s*) between species, it is more likely that they reflect differences in N*_e_*. For example, a lower long term N*_e_* in humans would allow more deleterious mutations to segregate at higher frequencies than in either mice or Drosophila. ML estimates of the demographic parameters of the model using four-fold sites as the neutral reference imply that there has been a recent increase in N*_e_* in *M. m. castaneus* (Table S3), as well as African *D. melanogaster* [32].

**Table 3:** Estimated percentages of amino acid mutations in different *N_e_s* ranges and estimates of *α*, the fraction of substitutions driven to fixation by positive selection.

Neutral Reference	Dataset	% of mutations in *N_e_s* range [95% CI]			*α* [95% CI]	*P*
		0–1	1–10	>10		
4-fold Sites	*M. m. castaneus*	10 [3/18]	11 [5/17]	79 [71/90]	0.57 [0.30/0.76]	–
	Human, EGP [28]	21 [16/28]	12 [6/18]	67 [60/73]	0.13 [−0.18/0.37]	0.014
	Human, EGP (subset)	22 [14/33]	12 [3/25]	66 [49/77]	−0.045 [−0.73/0.36]	0.014
	Human, PGA [33]	25 [19/32]	15 [7/23]	60 [53/66]	0.31 [0.055/0.50]	0.11
	Human [20]	21 [20/24]	12 [10/14]	66 [66/67]	0.21 [0.12/0.27]	0.020
	*D. melanogaster* [11]	6 [4/7]	7 [5/9]	87 [85/89]	0.52 [0.39/0.62]	0.54
Intronic Sites	*M. m. castaneus*	14 [6/23]	10 [3/15]	76 [70/82]	0.45 [0.063/0.71]	–
	Human, EGP [28]	27 [19/36]	13 [4/20]	60 [53/67]	0.034 [−0.36/0.34]	0.11
	Human, EGP (subset)	32 [21/40]	4 [3/15]	64 [53/72]	−0.44 [−0.85/0.13]	0.006
	Human, PGA [33]	32 [25/40]	14 [7/22]	53 [47/59]	0.13 [−0.11/0.34]	0.16

Estiamtes are obtained assuming either 4-fold degenerate sites or intronic sites as the neutral reference. P-values correspond to the comparison of *α* for each species (other than *M. m. castaneus*) with *M. m. castaneus*. Data analysed are: *M. m. castaneus*, this study, contrasted with *M. famulus*. African EGP polymorphism data set [28] contrasted with macaque. EGP subset refers to the set of gene orthologs sequenced in *M. m. castaneus*. African PGA data set [33] contrasted with macaque. African American population polymorphism data from Boyko et al. [20] contrasted with chimpanzee. African *D. melanogaster* polymorphism data [11] contrasted with *D. simulans*. The fitnesses of the wild-type, heterozygote, and mutant homozygote genotypes are assumed to be 1, 1−*s*/2, and 1−*s*, respectively. 95% confidence intervals are shown in square brackets.

**Table 4:** P-values for contrast between estimated frequencies of nearly neutral (*N_e_s*<1) mutations and strongly deleterious (*N_e_s*>10) mutations between *M. m. castaneus* and human datasets.

Neutral Reference	Dataset	P-value for mutation frequency class contrasted	
		*N_e_s<1*	*N_e_s>10*
4-folds	EGP [28]	0.02	0.026
	EGP (subset)	0.038	0.068
	PGA [33]	0.01	0.002
	Boyko et al. [20]	0.014	0.008
Introns	EGP [28]	0.036	0.002
	EGP (subset)	0.02	0.028
	PGA [33]	0.006	<0.002

P-values are calcualted seperately for analysis involving 4-fold degenerate synonymous sites or introns as the neutral reference.

A lack of neutral diversity in fast evolving genes has previously been interpreted as evidence for the effects of selective sweeps and therefore adaptation in Drosophila [13],[34],[35]. However, in contrast to these results, we found a nonsignificant positive correlation between synonymous site diversity and nonsynonymous divergence (Spearman *r* = 0.21, *p* = 0.084 for *d*_N_ vs. *θ_π_* and *r* = 0.16, *p* = 0.084 for *d*_N_ vs *θ_S_*). Therefore, unlike in Drosophila, our data do not suggest that selective sweeps in genes undergoing high rates of adaptive evolution reduce local neutral diversity, although our relatively small data set limits the power of this analysis.

To further investigate evidence for adaptation we estimated the fraction of adaptive amino acid substitutions, *α*, between *M. m. castaneus* and either *M. famulus* or the rat by a method related to the McDonald-Kreitman test for adaptive evolution [7] that contrasts polymorphism with divergence [22]. The method attempts to account for nearly neutral amino acid mutations, which, when compared to strongly deleterious mutations, contribute proportionately more to polymorphism than divergence. The parameters of the distribution of effects of deleterious amino acid mutations, estimated by ML from polymorphism within *M. m. castaneus*, and the neutral site divergence between *M. m. castaneus* and the outgroup (*M. famulus* or rat) are used to compute the number of amino acid substitutions expected between *M. m. castaneus* and the outgroup. The estimated fraction of adaptive substitutions is the difference between this expected number and the observed number of amino acid substitutions, scaled by the observed number (see [22] and Methods). Simulations suggest that the method produces close to unbiased estimates of *α* if the assumptions of the model are met, and is robust to substantial departures from the model assumptions, including complex demographic scenarios and linkage between sites [22]. However, in common with all McDonald-Kreitman based approaches, it is sensitive to long-term population size changes, a point that is discussed later.

Our estimate of *α* for wild mice, assuming four-fold sites as the neutral reference, is 57% (Table 3). This is somewhat higher than, but non-significantly different to, an estimate of 52% for African *D. melanogaster* with divergence to *D. simulans* (*P* = 0.54). However, the estimate for *α* in mice is very much higher than estimates for hominids for all the human polymorphism data sets, including the EGP data (*P* = 0.014 for a comparison only involving the subset of gene orthologs sequenced in mice using divergence to macaque) and PGA (*P* = 0.11 using divergence to macaque), and the data set of Boyko et al. [20] (*P* = 0.020 using divergence to chimpanzee). CpG dinucleotides have an elevated mutation rate in mammals and differ in frequency between coding and non-coding DNA. However, using only sites that are unlikely to be part of a CpG dinucleotide (non-CpG-prone sites) yields estimates of *α* that are similar to those based on all sites (Table 5).

**Table 5:** Estimates of the fraction of substitutions driven to fixation by positive selection obtained from estimates based on the inferred distribution of effects.

Site type	Outgroup	Neutral reference	*α* [95% CI]
All	*M. famulus*	4-fold	0.57 [0.30/0.76]
		intron	0.45 [0.063/0.71]
	Rat	4-fold	0.44 [0.13/0.69]
		intron	0.33 [−0.11/0.68]
Non-CpG-prone	*M. famulus*	4-fold	0.54 [0.0036/0.76]
		intron	0.56 [−0.12/0.88]
	Rat	4-fold	0.37 [−0.36/0.65]
		intron	0.51 [−0.044/0.86]

Estiamtes are calculated for different classes of sites and using either rat and *M. famulus* as outgroups. 95% confidence intervals are shown in square brackets.

**Figure 2:**
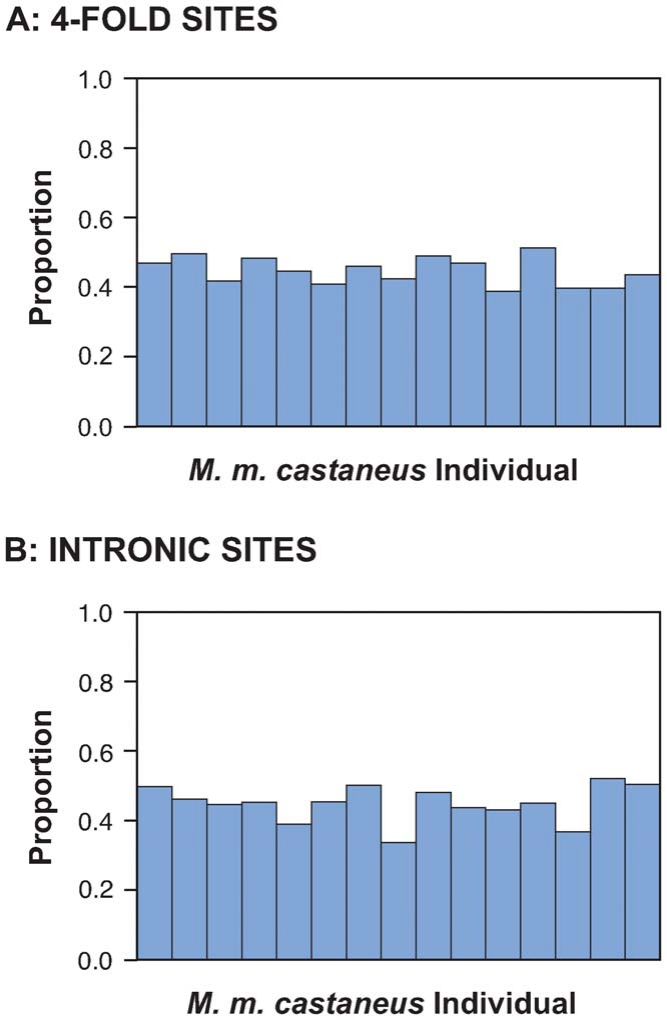
Proportion of ancestry assigned to population 1. Assuming *K* = 2 using 82 (4-fold degenerate sites, (A)) or using 84 (intronic sites, (B)) unlinked SNP loci. Each column represents one of the 15 *M. m. castaneus* individuals.

Estimates of *α* could also be affected by more complex demographic scenarios, such as admixture between differentiated sub-species and/or population subdivision, that are not modelled in our algorithm. We tested for evidence of population structure or admixture using the program *Structure* [36] using one randomly sampled four-fold degenerate or intronic SNP per sequenced locus. For both intronic and 4-fold degenerate synonymous sites we found no evidence for population subdivision in the *M. m. castaneus* sample, since the “no-admixture” model gives *P* = 1 in all but one case for a number of populations parameter K = 1 (see Table S4 for details). However, under the “admixture” model there is better support for two populations (K = 2) than one population (Table S4), suggesting population subdivision. Figure 2 shows an ancestry plot for one randomly selected run that provided support for K = 2. In this plot each individual shows ancestries in both putative populations (with roughly equal proportions in the population), suggesting that they are admixed. However, we do not find any individuals that have ancestries in just one of the two populations (i.e., there are no individuals that are purely from population 1 or purely from population 2), suggesting that this result can be explained by a violation of an assumption in *Structure*, namely that genotype frequencies are at Hardy-Weinberg equilibrium. House mice are known to inbreed in the wild [37], potentially causing an elevated inbreeding coefficient (*F_is_*). To determine whether such an effect can be observed in our sample, we calculated *F_is_* values [38] for each SNP using the program *Genepop* (http://genepop.curtin.edu.au/). We found a substantial excess of loci showing positive *F_is_* values, indicating a deficiency of heterozygotes and a deficiency of negative *F_is_* values, indicating an excess of homozygotes (Figure S1). Thus, our sample shows evidence for inbreeding. In summary, our interpretation of these results is that the *M. m. castaneus* population shows no evidence for hidden population substructure or admixture between differentiated subspecies, but there is evidence that inbreeding is a feature of all individuals used in the study.

We attempted to account for the effect of inbreeding, and therefore the possibility that alleles from the same individual are not independent samples from the population, by repeating the analysis for 20 datasets created by randomly selecting one allele from each individual for each site, such that each sequence analysed in each data set was a composite, derived from a single individual. We then calculated mean estimates of *α* by averaging over the 20 randomly generated datasets. When calculated by this method, our estimates of mean *α* are only marginally lower than estimates using the complete data set (see Table S5).

Estimates of *α* obtained using rat as the outgroup are generally somewhat lower than those using *M. famulus*, but are still close to 40% (estimates range from 0.33 to 0.51, see Table 5), suggesting that the estimate of *α* in murids is robust to the choice of outgroup. An earlier method to estimate *α* [8] attempts to remove the influence of nearly neutral deleterious mutations by excluding polymorphisms at a frequency below an arbitrary threshold (e.g., 10%). Estimates of *α* produced by this method are somewhat lower than the estimates from our method (Table 6), but this is expected because estimates are likely to be downwardly biased [21]. However, they are substantially higher than estimates of *α* using this method in hominids [8]. If all sites are included in this analysis, irrespective of their frequency, estimates of *α* in wild mice are close to zero, or even negative (Table 6). This is likely to be due to slightly deleterious mutations, which contribute low frequency polymorphisms but have little chance of fixation, and lead to downwardly biased *α* estimates. Indeed even when low-frequency, segregating at a frequency of <10%, are excluded, analyses suggest that estimates of *α* may still be downwardly biased.

**Table 6:** Estimates of the fraction of substitutions driven to fixation by positive selection obtained using a simple extension of the McDonald-Kreitman test [7].

Site type	Outgroup	Neutral reference	*α*_FWW_ [SE]	*α*_FWW_>10% [SE]
All	*M. famulus*	4-fold	0.14 [0.13]	0.35 [0.15]
		intron	0.0020 [0.18]	0.21 [0.22]
	Rat	4-fold	−0.067 [0.14]	0.22 [0.14]
		intron	−0.22 [0.16]	0.076 [0.18]
Non-CpG-prone	*M. famulus*	4-fold	0.081 [0.23]	0.40 [0.23]
		intron	−0.077 [0.26]	0.44 [0.27]
	Rat	4-fold	−0.094 [0.19]	0.18 [0.24]
		intron	−0.13 [0.21]	0.26 [0.26]

Estimates are calculated using all sites (*α*_FWW_) and using only sites with variants >10% (*α*_FWW_>10%) for different classes of sites and using either rat and *M. famulus* as outgroups. Standard errors are shown in square brackets.

Currently available estimates of *α* from a variety of species vary widely. The estimates of the fractions of adaptive substitutions in microbes [10], Drosophila [9],[11],[12] and now mice present a serious challenge to the neutralist view of protein evolution. Taken together these results suggest that most amino acid substitutions are caused by positive selection, and that genetic drift is therefore not the most important cause of protein evolution. However, estimates of *α* obtained for yeast [14],[15], Arabidopsis [16], and hominids (which are ∼10% at most [17]–[20]) suggest the opposite. There are several possible explanations for these discrepancies. One possibility is that the estimates obtained for yeast and Arabidopsis are not based on an explicit population genetics model, and even though attempts have been made to reduce the impact of slightly deleterious mutations, the estimates may still be downwardly biased. Nevertheless, these results are hard to reconcile with estimates from microbes, Drosophila and mice, since both of these species are thought to have a relatively high N*_e_*. On the other hand, the low estimated proportion of adaptive substitutions in hominids may reflect their low N*_e_*, since this will increase the proportion of effectively neutral advantageous and deleterious mutations. Low N*_e_* will also reduce the rate of adaptive evolution if the rate is limited by the supply of mutations. This is consistent with the low recent N*_e_* estimates for humans [23], chimpanzees [39] and gorillas [40]. It is also possible that most adaptive evolution occurs in noncoding regions in primates [41].

Alternatively, changes in effective population size can lead to bias in the estimate of *α* [7],[42]. It can be shown that if the true value of *α* is independent of N*_e_*, but that the current N*_e_* (which affects the level of polymorphism) is different to the average N*_e_* over the evolution of the species (which affects the level of divergence) then the relationship between the true (*α_true_*) and estimated (*α_est_*) values of *α* is given by
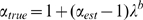
(1)
[22], if the distribution of fitness effects is gamma, where λ is the ratio of the current and ancestral N*_e_* and *b* is the shape parameter of the gamma distribution of mutational effects. Thus, a contraction in N*_e_* will lead to an underestimate of *α* and an increase will lead to an overestimate. It is therefore possible that the difference in the estimate of *α* between hominids and rodents is due to recent demography; if the current N*_e_* of humans was much smaller than the ancestral population size, and/or the current N*_e_* of *M. m. castaneus* was much larger than the ancestral, then *α_true_* could be very similar in the two species. Recent evidence suggests that ancestral great ape N*_e_* may have been substantially bigger than current [43]. So, for example, assuming *b* for humans is 0.2 [20],[32],[44], *α_est_* = 0.1 implies *α_true_* = 0.35 and 0.43 for 5- and 10-fold reductions in long-term N*_e_*, respectively (equation 1). However, we also infer from our polymorphism data that *M. m. castaneus* has undergone a recent increase in N*_e_*, although our evidence for this is modest. Nevertheless, assuming that current *M. m. castaneus *N*_e_* is 5- and 10-fold larger than the ancestral N*_e_*, our estimates of *α_est_* = 0.57 and *b* = 0.31 (see Table S3) would imply that *α_true_* = 0.29 and 0.12 respectively (consistent with the estimates from humans). More estimates of *α* from other murid and mammalian species will help to determine whether the high rate of adaptive evolution we have inferred is widespread amongst murid species and therefore not an artefact of demography.

## Materials and Methods

### Sampling of wild mice

The 15 *M. m. castaneus* individuals were collected in 2003 in 4 localities (2–5 individuals per locality) along a 130 km transect (from lat 32.244987°, lon 77.188181° to lat 30.977139°, lon 76.986026°) south of the Himalayas in the North-West Indian state of Himachal Pradesh. Each locality extended over an area covering 5 km^2^. To avoid collecting related individuals, we analysed only one individual per trap site within each locality and trap sites had to be separated by >500m. An individual *M. famulus*, originating from India (locality Kotagiri), was obtained from the Montpellier wild mice genetic repository (http://www.isem.cnrs.fr/spip.php?article477).

### Selection of amplicons

DNA sequences were generated for genes sampled from a set whose human orthologs have been sequenced in the Environmental Genome Project (EGP) [28]. All genes sequenced as part of EGP are autosomal and many had polymorphism data available for African human populations at the time of searching, allowing us to make a direct comparison of the results we obtained in mice with results based on the same set of genes in a human population. Loci sequenced in Africans (618 as of 7th August 2007) whose orthologs could be identified in the mouse genome (585 genes, using NCBI Homologene) were considered. For 77 loci, DNA sequences were generated for the 15 *M. m. castaneus* individuals and one *M. famulus* individual. Primers were designed to amplify regions that captured coding and intronic DNA using Primer3 [45].

### DNA sequences

PCR reactions were performed using GoTaq DNA polymerase (Promega) using a touchdown program consisting of 95°C for 15 minutes, followed by 28 cycles of 95°C for 30 seconds, 62°C for 45 seconds (reducing by 0.5°C every cycle), 72°C for 2 minutes, then 12 cycles of 95°C for 30 seconds, 52 °C for 45 seconds and 72°C for 2 minutes, with a final extension at 72°C for 10 minutes. Following evaluation on 1% agarose gels, products were purified using ExoSAP-IT (USB), or, if product indicated non-specific priming, the appropriate band was cut from a gel and extracted using Qiaquick gel extraction kit (Qiagen). Forward and reverse sequences were generated using Big Dye Terminator Sequencing Kits (Applied Biosystems) on an ABI Prism 3730 DNA Analyzer.

Sequence analysis and variant detection was carried out using CodonCode Aligner version 2.0.6 (http://www.codoncode.com/aligner/). Sequences had an average Phred score of >60. All sequence traces were manually checked. CodonCode was set to highlight any site with a Phred score <30 (which could include low quality sequence or heterozygous sites). All such sites were manually checked, but in order to avoid excluding heterozygotes, were not automatically excluded. Alignments between the 15 *M. m. castaneus*, the *M. famulus* individual and the *M. m. musculus* reference sequence were obtained. All alignments were manually checked before further analysis.

#### Processing of sequence data

Orthologous *Rattus norvegicus* sequences were obtained for each amplicon using a combination of approaches. Initially, the mouse reference sequence was BLASTed [46] against two different assemblies of the rat genome, downloaded from UCSC genome browser. The first was produced by the Baylor College of Medicine Human Genome Sequencing Center (BCM-HGSC) as part of the Rat Genome Sequencing Consortium, and the second (referred to as the alternative assembly) was produced by Celera Genomics. If no reciprocal-best-hit was found in the standard assembly, a reciprocal-best-hit search was done in the alternative assembly. The mouse and rat sequences were then aligned using MAVID [47], the alignments checked by eye, and poorly aligned sections masked in rat. If the reciprocal-best-hits approach failed to identify an orthologous section in rat, the relevant section from the “multiz30way” whole genome sequence alignments of 30 vertebrates (available at the UCSC genome browser http://genome.ucsc.edu/) was checked. If the sequence of interest was located entirely within a single unbroken alignment for mouse and rat, then the rat sequence was considered as orthologous, and the relevant section of the alignment was realigned, checked and masked as before. Using this procedure, we successfully identified rat sequences orthologous to at least part of each mouse amplicon.

Alignments for each amplicon were generated between *M. m. musculus* release mm9, the *M. m. castaneus* individuals, *M. famulus* and rat. Then, using the *M. m. musculus* annotation, sites were categorised as “0-fold” (0-fold degenerate sites, or nonsynonymous), “2-fold” (2-fold degenerate sites), “4-fold” (4-fold degenerate synonymous sites, or synonymous) or “intron”. We excluded potential splice sites (defined as the first 6bp or last 16bp of an intron). Sites were also scored for their CpG-prone status (defined as being preceded by a C or followed by a G in any species).

#### Summary statistics

We calculated two standard estimates of diversity, *θ_S_*, the number of segregating sites divided by a normalising factor 
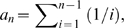
 where n is the number of sequences [48], and *θ_π_*, the average heterozygosity, also known as nucleotide diversity or π [49]. Both estimators, when normalised per site, are unbiased estimates of 4N*_e_μ* (under a Fisher-Wright neutral of drift and an infinite sites model of mutation, such that each site experiences no more than one mutation). However, they are expected to differ if there is an excess or deficit of low or high frequency variants. This difference and therefore the level of skew in the SFS can be quantified with Tajima's *D* [29]. However, since *D* can only be calculated when the same number of chromosomes has been sequenced at each site (and this is not the case in the data presented here, see Table S6), we sampled sequences 20 times without replacement at each site (rejecting sites with fewer than 20 sequences) such that the number of chromosomes sampled per site was constant. We also calculated divergence between the *M. m. castaneus* sequences and both *M. famulus* and rat sequences using a Jukes-Cantor correction for multiple hits. 95% confidence intervals for *θ_π_*, *θ_S_*, Tajima's *D* and divergence were calculated by bootstrapping 1,000 times by locus.

### Inference of the distribution of fitness effects of new amino acid mutations

We extended a maximum likelihood approach [32] to estimate parameters of the distribution of fitness effects of new amino acid mutations using the allele frequency distributions (the site frequency spectra, SFSs) for 0-fold and putatively neutrally evolving sites (either 4-fold or intronic sites). We assumed that effects (*s*) of amino acid mutations are unconditionally deleterious, and sampled from a gamma distribution with shape and scale parameters *a* and *b*, respectively. These parameters were estimated along with the fraction of unmutated sites, *f*_0_, and demographic parameters N*_1_*, N*_2_* and *t*, corresponding to ancestral population size, current population size and the number of generations since a population size change, respectively. Polymorphism data were summed across loci using folded SFSs. We extended the method to allow variation in the number of alleles at each site. We generated SFSs for sites with the same numbers of alleles, computed the log likelihood for each SFS, and summed these to compute the overall log likelihood. Selective effects are estimated on a scale N*s*, where N is a measure of the population size at the time that the polymorphism data are censured. Under the assumption of a single step change in population size, there may be little information to estimate the relative values of the population size before (N*_1_*) and after (N*_2_*) the size change if, for example, *t*≫N*_2_* or *t*≪N*_2_*. We therefore computed a weighted recent population size from
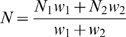
where 
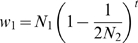
 and 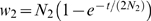.

### Estimating the proportion of adaptive substitutions, *α*

We estimated *α* for 0-fold substitutions using a method that attempts to account for the segregation of slightly deleterious mutations and recent population size changes [22]. The divergence at neutrally evolving sites (*d_S_*) is proportional to the mutation rate per site. At selectively evolving sites, the expected divergence due to fixation of deleterious mutations is proportional to the product of the mutation rate and the fixation probability, *u*(N, N*_e_s*), of a new mutation [50]. Defining *d*_N_ as the observed divergence at the selectively evolving sites, and equating N with N*_e_* (because these are equivalent under the transition matrix method under which population size is estimated) *α* is proportional to the difference between the observed and expected divergence:
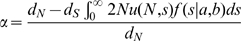
(note that *d_S_* and *ds* are different quantities). *α* was initially estimated using all sequenced alleles (*i.e.* a total of 30 alleles, two per individual).

To test whether our estimates of alpha could be biased as a result of assuming that different alleles from the same individual are independent, we also estimated *α* using only a selected and neutral reference sequence for each individual. Specifically we created 20 data sets in which, for each individual and for every site, we randomly picked a single base from the individuals' two alleles. Each data set therefore consisted of up to 15 selected/neutral reference sequences, each one corresponding to a different individual. Mean estimates of *α* were then computed by averaging over the 20 randomly generated datasets.

We also used Fay, Wyckoff and Wu's [8],[9] extension of the McDonald-Kreitman test to estimate the fraction of 0-fold substitutions driven to fixation by positive selection, *α_FWW_*:
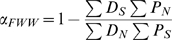
where *D*_N_ (*P*_N_) and *D_S_* (*P_S_*) are numbers of divergent (polymorphic) sites for selected and neutral classes, respectively, and the summation is over genes. To reduce the influence of nearly neutral alleles on *α_FWW_*, we excluded sites where the rare variant was at a frequency of 10% or less. This method assumes that the number of alleles sampled is constant, so we again sampled the alignments to give 20 alleles per site and ignored sites that lacked an orthologous base in the outgroup. 95% confidence intervals for all statistics were obtained by bootstrapping 1,000 times by locus.

### Inference of population structure

House mice, especially those originating from the ancestral range, could exhibit a complex genetic composition, reflecting either incomplete lineage sorting or admixture between the different subspecies [30],[51]. We used our multilocus SNP dataset to determine if the population sample of *M. m. castaneus* that was used in our study shows evidence for admixture or hidden population substructure. We randomly selected one SNP from each amplicon, excluding SNPs that cover known splice sites and only including SNPs where at least 10 of the 15 individuals could be sequenced. We excluded any SNPs covering indel sites. We separately analysed SNPs from intronic sites and SNPs from 4-fold degenerate sites. Altogether 84 (intronic data) or 82 (4-fold degenerate sites) unlinked SNP loci were included in the analysis.

We used the program *Structure* [36] to identify the presence of different subpopulations in the sample, if any, and to estimate the ancestry of the sampled individuals in each of these subpopulations. The number of subpopulations is inferred by calculating the probability P(*X*|K) of the data given a certain prior value of K (number of subpopulations) over a number of Monte Carlo Markov Chain (MCMC) iterations. The posterior probabilities P(K|*X*) can be calculated following Bayes' rule. The subpopulations are characterised by different allele frequencies, and, according to their multilocus genotypes, individuals are probabilistically assigned to one or more subpopulations. The scores of individuals in the subpopulations correspond to the probability of ancestry in any one of them. In this study we assumed prior values of K from 1 to 4. We considered two models for the ancestry of individuals. In the first, the “no-admixture model”, individuals are assumed to be drawn purely from one of K populations. In the second, the “admixture model”, individuals are allowed to have mixed ancestry, that is, some fraction of an individual's genome comes from different subpopulations. Both of those models assume that all the markers are unlinked and provide independent information on an individual's ancestry. Inferences of the number of subpopulations and ancestries of individuals are based on 1,000,000 iterations of the MCMC, after a “burn-in” period of 100,000 iterations. We ran the program without incorporation of prior population information. We performed 3 independent runs of the Markov chain for each parameter set to check for convergence of the chains.

## Supporting Information

Figure S1 Distribution of *F_is_* values calculated for individual SNP loci for 4-fold degenerate synonymous and intronic sites.
(0.34 MB EPS)

Table S1 Details of the 77 genes selected for sequencing. Reported are gene names, gene IDs with NCBI Entrez Gene for humans and the mouse ortholog, as well as the Ensembl ID for the mouse ortholog.
(0.11 MB DOC)

Table S2 Estimates of percentage diversity (*θ_π_* and *θ_S_*) summed over all sites for *M. m. castaneus*, and estimates of divergence (*d*) to *M. famulus* or the rat for non-CpG-prone sites only. Standard Errors are shown in square brackets.
(0.03 MB DOC)

Table S3 Demographic parameter estimates along with estimates of the shape parameter from the gamma distribution (*b*). Estimates are calculated using either 4-fold degenerate synonymous sites or intronic sites as the neutral standard, for all sites and non-CpG-prone only sites.
(0.03 MB DOC)

Table S4 Analysis of population structure in *M. m. castaneus* from Northern India. Rows with the most probable number of sub-populations given the data in each run are highlighted in yellow.
(0.07 MB DOC)

Table S5 Estimates of the fraction of substitutions driven to fixation by positive selection obtained using only a single allele from each individual. Mean estimates of α were computed by averaging over the results from 20 randomly generated datasets, where each data set contains a single sequence for each individual constructed by sampling a single base from the individuals' two alleles at every site. Calculations are performed using two different classes of sites, both rat and *M. famulus* as outgroups and using both 4-fold degenerate synonymous sites and intron sites as the neutral reference.
(0.04 MB DOC)

Table S6 Summary of coverage for the different sequence classes. Reported are the number of loci sequenced for each class, the total number of sites covered by the amplicons (no. sites), the fraction of sites with complete coverage of 30 alleles across all loci (30), the fraction of sites with at least 25 alleles sampled (>25) and the mean coverage per site per individual (Mean).
(0.04 MB DOC)
