# Supplementary material for: Evidence for Pervasive Adaptive Protein Evolution in Wild Mice
Source: PLoS Genet. 2010 Jan 22;6(1):e1000825. doi: 10.1371/journal.pgen.1000825 (PMC2809770; doi:10.1371/journal.pgen.1000825)
Supplement: Table S1 — Details of the 77 genes selected for sequencing. Reported are gene names, gene IDs with NCBI Entrez Gene for humans and the mouse ortholog, as well as the Ensembl ID for the mouse ortholog. (0.11 MB DOC) [file pgen.1000825.s002.doc]

**Table S1 - Details of the 77 genes selected for sequencing.**

| Name | Human Entrez Gene ID | Mouse Entrez Gene ID | Mouse ensembl ID |
| --- | --- | --- | --- |
| Pla2g4a | 5321 | 18783 | ENSMUSG00000056220 |
| Umps | 7372 | 22247 | ENSMUSG00000022814 |
| Egfr | 1956 | 13649 | ENSMUSG00000020122 |
| Orc3l | 23595 | 50793 | ENSMUSG00000040044 |
| Ela1 | 1990 | 109901 | ENSMUSG00000023031 |
| Cyp2c39 | 1557 | 13098 | ENSMUSG00000025003 |
| Nf1 | 4763 | 18015 | ENSMUSG00000020716 |
| Xpc | 7508 | 22591 | ENSMUSG00000030094 |
| Angptl7 | 10218 | 654812 | ENSMUSG00000028989 |
| prkcm | 5587 | 18760 | ENSMUSG00000002688 |
| mmp21 | 118856 | 214766 | ENSMUSG00000030981 |
| mad2l2 | 10459 | 71890 | ENSMUSG00000029003 |
| prdx1 | 5052 | 18477 | ENSMUSG00000028691 |
| bace1 | 23621 | 23821 | ENSMUSG00000032086 |
| Cyp2b10 | 1555 | 13088 | ENSMUSG00000030483 |
| cdc20 | 991 | 107995 | ENSMUSG00000006398 |
| fgfr2 | 2263 | 14183 | ENSMUSG00000030849 |
| Mmp15 | 4324 | 17388 | ENSMUSG00000031790 |
| Cyp17a1 | 1586 | 13074 | ENSMUSG00000003555 |
| Adm | 133 | 11535 | ENSMUSG00000030790 |
| Tert | 7015 | 21752 | ENSMUSG00000021611 |
| Cyp4b1 | 1580 | 13120 | ENSMUSG00000028713 |
| Rrm2b | 50484 | 382985 | ENSMUSG00000022292 |
| prdx2 | 7001 | 21672 | ENSMUSG00000005161 |
| mt3 | 4504 | 17751 | ENSMUSG00000031760 |
| mms19l | 64210 | 72199 | ENSMUSG00000025159 |
| mpo | 4353 | 17523 | ENSMUSG00000009350 |
| Epx | 8288 | 13861 | ENSMUSG00000052234 |
| tgfb1i1 | 7041 | 21804 | ENSMUSG00000030782 |
| mapk11 | 5600 | 19094 | ENSMUSG00000053137 |
| Cyp1a1 | 1543 | 13076 | ENSMUSG00000032315 |
| stk25 | 10494 | 59041 | ENSMUSG00000026277 |
| csk | 1445 | 12988 | ENSMUSG00000032312 |
| por | 5447 | 18984 | ENSMUSG00000005514 |
| mmp16 | 4325 | 17389 | ENSMUSG00000028226 |
| rev1 | 51455 | 56210 | ENSMUSG00000026082 |
| adh1 | 126 | 11522 | ENSMUSG00000074207 |
| adh4 | 127 | 26876 | ENSMUSG00000037797 |
| adh5 | 128 | 11532 | ENSMUSG00000028138 |
| itgal | 3683 | 16408 | ENSMUSG00000030830 |
| tpo | 7173 | 22018 | ENSMUSG00000020673 |
| Capn3 | 825 | 12335 | ENSMUST00000028748 |
| ddb1 | 1642 | 13194 | ENSMUST00000025649 |
| pdlim1 | 9124 | 54132 | ENSMUSG00000055044 |
| tjp1 | 7082 | 21872 | ENSMUSG00000030516 |
| ppib | 5479 | 19035 | ENSMUST00000034947 |
| cdc42bpb | 9578 | 217866 | ENSMUSG00000021279 |
| aoc2 | 314 | 237940 | ENSMUST00000041095 |
| aoc3 | 8639 | 11754 | ENSMUST00000103105 |
| ckb | 1152 | 12709 | ENSMUST00000001304 |
| Nos2 | 4843 | 18126 | ENSMUSG00000020826 |
| fmo3 | 2328 | 14262 | ENSMUST00000028010 |
| tdp1 | 55775 | 104884 | ENSMUST00000021594 |
| gab1 | 2549 | 14388 | ENSMUSG00000031714 |
| abp1 | 26 | 76507 | ENSMUSG00000029811 |
| poln | 353497 | 272158 | ENSMUST00000042954 |
| fgf21 | 26291 | 56636 | ENSMUST00000033099 |
| blm | 641 | 12144 | ENSMUST00000081314 |
| hspa5 | 3309 | 14828 | ENSMUST00000028222 |
| msr1 | 4481 | 20288 | ENSMUST00000026021 |
| pax3 | 5077 | 18505 | ENSMUST00000004994 |
| abl1 | 25 | 11350 | ENSMUST00000028190 |
| recql4 | 9401 | 79456 | ENSMUST00000036852 |
| casp8 | 841 | 12370 | ENSMUST00000027189 |
| osr1 | 130497 | 23967 | ENSMUST00000057021 |
| rxra | 6256 | 20181 | ENSMUST00000077257 |
| dclre1b | 64858 | 140917 | ENSMUSG00000027845 |
| cdc37 | 11140 | 12539 | ENSMUSG00000019471 |
| neil3 | 55247 | 234258 | ENSMUST00000047768 |
| foxm1 | 2305 | 14235 | ENSMUSG00000001517 |
| fmo5 | 2330 | 14263 | ENSMUSG00000028088 |
| scara3 | 51435 | 219151 | ENSMUSG00000034463 |
| sprr3 | 6707 | 20766 | ENSMUSG00000045539 |
| sepp1 | 6414 | 20363 | ENSMUSG00000064373 |
| birc2 | 329 | 11797 | ENSMUSG00000057367 |
| fgf5 | 2250 | 14176 | ENSMUSG00000029337 |
| chrna4 | 1137 | 11438 | ENSMUSG00000027577 |

Reported are gene names, gene IDs with NCBI Entrez Gene for humans and the mouse ortholog as well as the Ensembl ID for the mouse ortholog.
