# Supplementary material for: Evidence for Pervasive Adaptive Protein Evolution in Wild Mice
Source: PLoS Genet. 2010 Jan 22;6(1):e1000825. doi: 10.1371/journal.pgen.1000825 (PMC2809770; doi:10.1371/journal.pgen.1000825)
Supplement: Table S2 — Estimates of percentage diversity (θπ and θS) summed over all sites for M. m. castaneus, and estimates of divergence (d) to M. famulus or the rat for non-CpG-prone sites only. Standard Errors are shown in square brackets. (0.03 MB DOC) [file pgen.1000825.s003.doc]

**Table S2** - **Estimates of percentage diversity (*θ_π_* and *θ_S_*) summed over all sites for *M. m. castaneus*, and estimates of divergence (*d*) to *M. famulus* or the rat for non-CpG-prone sites only.**

| Site class | *% θ_π_* [SE] | *% θ_S_* [SE] | Tajima's *D* [SE] | *% d* (*M. famulus*) [SE] | *% d* (rat) [SE] |
| --- | --- | --- | --- | --- | --- |
| 0-fold | 0.14 [0.022] | 0.20 [0.022] | -0.90 [0.27] | 0.79 [0.12] | 3.5 [0.42] |
| 2-fold | 0.48 [0.060] | 0.63 [0.060] | -1.0 [0.24] | 2.3 [0.26] | 12 [0.61] |
| 4-fold | 0.77 [0.12] | 0.83 [0.12] | -0.26 [0.29] | 3.3 [0.27] | 19 [0.80] |
| Intron | 0.61 [0.051] | 0.76 [0.051] | -0.80 [0.10] | 2.8 [0.15] | 15 [0.49] |

Standard Errors are shown in square brackets.
