# Supplementary material for: Evidence for Pervasive Adaptive Protein Evolution in Wild Mice
Source: PLoS Genet. 2010 Jan 22;6(1):e1000825. doi: 10.1371/journal.pgen.1000825 (PMC2809770; doi:10.1371/journal.pgen.1000825)
Supplement: Table S3 — Demographic parameter estimates along with estimates of the shape parameter from the gamma distribution (b). Estimates are calculated using either 4-fold degenerate synonymous sites or intronic sites as the neutral standard, for all sites and non-CpG-prone only sites. (0.03 MB DOC) [file pgen.1000825.s004.doc]

**Table S3 - Demographic parameter estimates along with estimates of the shape parameter from the gamma distribution (*b*)**.

| Site type | Neutral Reference | *N_2_/N_1_* | *t/N_2_* | *b* |
| --- | --- | --- | --- | --- |
| All | 4-fold | 2.31 | 0.166 | 0.314 |
|  | Intronic | 4.09 | 0.643 | 0.236 |
| Non-CpG-prone | 4-fold | 10 | 2.98 | 0.288 |
|  | intron | 10 | 1.12 | 0.362 |

Estimates are calculated using either 4-fold degenerate synonymous sites or intronic sites as the neutral standard, for all sites and non-CpG-prone only sites.
