# Supplementary material for: Evidence for Pervasive Adaptive Protein Evolution in Wild Mice
Source: PLoS Genet. 2010 Jan 22;6(1):e1000825. doi: 10.1371/journal.pgen.1000825 (PMC2809770; doi:10.1371/journal.pgen.1000825)
Supplement: Table S4 — Analysis of population structure in M. m. castaneus from Northern India. Rows with the most probable number of sub-populations given the data in each run are highlighted in yellow. (0.07 MB DOC) [file pgen.1000825.s005.doc]

**Table S4 - Analysis of population structure in *M. m. castaneus*** from Northern India.

| Run | Model | K | 4-fold Sites | | Intronic Sites | |
| --- | --- | --- | --- | --- | --- | --- |
|  |  |  | ln[P(*X*\|*K*)] | P(*K*\|*X*) | ln[P(*X*\|*K*)] | P(*K*\|*X*) |
| 1 | Admix | 1 | -794.2 | 0.01 | -811.2 | <0.001 |
|  | Admix | 2 | -789.8 | 0.99 | -804 | 1 |
|  | Admix | 3 | -824.2 | <0.001 | -870 | <0.001 |
|  | Admix | 4 | -1072.8 | <0.001 | -1073.9 | <0.001 |
| 2 | Admix | 1 | -794.8 | 0.00 | -810.1 | 0.00 |
|  | Admix | 2 | -789.4 | 1 | -804.7 | 1 |
|  | Admix | 3 | -806.2 | <0.001 | -826.2 | <0.001 |
|  | Admix | 4 | -816.9 | <0.001 | -1165.2 | <0.001 |
| 3 | Admix | 1 | -793.7 | 0.01 | -805.9 | 0.48 |
|  | Admix | 2 | -789.3 | 0.99 | -805.8 | 0.52 |
|  | Admix | 3 | -810.1 | <0.001 | -832.4 | <0.001 |
|  | Admix | 4 | -844.1 | <0.001 | -1063.5 | <0.001 |
| 1 | NoAdmix | 1 | -793.7 | 0.99 | -809.9 | 1 |
|  | NoAdmix | 2 | -880.6 | <0.001 | -972.6 | <0.001 |
|  | NoAdmix | 3 | -865.2 | <0.001 | -871.6 | <0.001 |
|  | NoAdmix | 4 | -798.8 | 0.01 | -824.8 | <0.001 |
| 2 | NoAdmix | 1 | -793.3 | 0.65 | -808.9 | 1 |
|  | NoAdmix | 2 | -866.6 | <0.001 | -921.8 | <0.001 |
|  | NoAdmix | 3 | -1052.1 | <0.001 | -860.2 | <0.001 |
|  | NoAdmix | 4 | -793.9 | 0.35 | -823.6 | <0.001 |
| 3 | NoAdmix | 1 | -794.6 | 1 | -810.6 | 1 |
|  | NoAdmix | 2 | -901.3 | <0.001 | -963.5 | <0.001 |
|  | NoAdmix | 3 | -847.7 | <0.001 | -904.3 | <0.001 |
|  | NoAdmix | 4 | -803.8 | 0.00 | -830.2 | <0.001 |

Rows with the most probable number of sub-populations given the data in each run are highlighted in yellow.
