# Supplementary material for: Evidence for Pervasive Adaptive Protein Evolution in Wild Mice
Source: PLoS Genet. 2010 Jan 22;6(1):e1000825. doi: 10.1371/journal.pgen.1000825 (PMC2809770; doi:10.1371/journal.pgen.1000825)
Supplement: Table S5 — Estimates of the fraction of substitutions driven to fixation by positive selection obtained using only a single allele from each individual. Mean estimates of α were computed by averaging over the results from 20 randomly generated datasets, where each data set contains a single sequence for each individual constructed by sampling a single base from the individuals' two alleles at every site. Calculations are performed using two different classes of sites, both rat and M. famulus as outgroups and using both 4-fold degenerate synonymous sites and intron sites as the neutral reference. (0.04 MB DOC) [file pgen.1000825.s006.doc]

**Table S5 - Estimates of the fraction of substitutions driven to fixation by positive selection obtained using only a single allele from each individual.**

| Site Class | Outgroup | Neutral Reference | *α* |
| --- | --- | --- | --- |
| All | *M. famulus* | 4-fold | 0.53 |
|  |  | intron | 0.33 |
|  | Rat | 4-fold | 0.38 |
|  |  | intron | 0.14 |
| Non-CpG-prone | *M. famulus* | 4-fold | 0.37 |
|  |  | intron | 0.48 |
|  | Rat | 4-fold | 0.23 |
|  |  | intron | 0.38 |

Mean estimates of α were computed by averaging over the results from 20 randomly generated datasets, where each data set contains a single sequence for each individual constructed by sampling a single base from the individuals’ two alleles at every site. Calculations are performed using two different classes of sites, both rat and *M. famulus* as outgroups and using both 4-fold degenerate synonymous sites and intron sites as the neutral reference.
