# Supplementary material for: Evidence for Pervasive Adaptive Protein Evolution in Wild Mice
Source: PLoS Genet. 2010 Jan 22;6(1):e1000825. doi: 10.1371/journal.pgen.1000825 (PMC2809770; doi:10.1371/journal.pgen.1000825)
Supplement: Table S6 — Summary of coverage for the different sequence classes. Reported are the number of loci sequenced for each class, the total number of sites covered by the amplicons (no. sites), the fraction of sites with complete coverage of 30 alleles across all loci (30), the fraction of sites with at least 25 alleles sampled (>25) and the mean coverage per site per individual (Mean). (0.04 MB DOC) [file pgen.1000825.s007.doc]

**Table S6 -** Summary of coverage for the different sequence classes.

| Site class | Loci | No. sites | 30 | >25 | Mean |
| --- | --- | --- | --- | --- | --- |
| 0-fold | 77 | 25,188 | 0.60 | 0.87 | 0.92 |
| 2-fold | 77 | 7,731 | 0.61 | 0.87 | 0.92 |
| 4-fold | 77 | 6,170 | 0.60 | 0.87 | 0.93 |
| intron | 60 | 28,169 | 0.40 | 0.65 | 0.85 |

Reported are the number of loci sequenced for each class, the total number of sites covered by the amplicons (no. sites), the fraction of sites with complete coverage of 30 alleles across all loci (30), the fraction of sites with at least 25 alleles sampled (>25) and the mean coverage per site per individual (Mean).
